# When technology awakens willingness but stalls action: the asymmetric psychological translation of managerial safety cognition

**DOI:** 10.3389/fpsyg.2026.1841993

**Published:** 2026-06-12

**Authors:** Bolu Wei, Shipeng Cui, Ru Guo, Xuemeng Guo, Mingrong Zeng, Chen Su

**Affiliations:** 1 China Academy of Safety Science and Technology, Beijing, China; 2 CNNP Rich Energy Guangdong Co., Ltd., Guangzhou, China; 3 Experimental College, Open University of China, Beijing, China; 4 School of Economics and Management, Beijing Jiaotong University, Beijing, China; 5 Research Centre, Ministry of Emergency Management, Beijing, China

**Keywords:** institutionalization, managerial safety cognition, multilevel analysis, performance pressure, safety motivation, safety participation, team safety climate, technological affordance

## Abstract

Why does managerial prioritization of safety more readily correspond to frontline willingness than to frontline action? Drawing on upper echelons theory, the attention-based view, and organizational psychology research on the attitude–behavior gap, this study examines whether managerial safety cognition is associated with frontline safety outcomes through three organizational mechanisms: institutionalization, technological affordance, and team safety climate. Using multi-source, time-lagged data from 183 firms and 667 nested teams in Chinese high-risk industries, this study estimated random-intercept multilevel models and used supervisor-rated behavior for cross-source validation. Managerial safety cognition was positively associated with all three organizational mechanisms. Institutionalization and team safety climate were, in turn, associated with both safety motivation and safety participation. Technological affordance was associated with safety motivation but not safety participation, and formal coefficient comparison tests confirmed that this asymmetry was statistically significant. Performance pressure further bounded behavioral translation in an inverted-U pattern consistent with the job demands–resources model: moderate pressure strengthened, whereas excessive pressure weakened, the behavioral relevance of organizational mechanisms. The findings support an attentional-prioritization account in which top-level safety priorities reach the frontline through differentiated organizational pathways rather than through a single leadership-signal channel. Practically, the findings suggest that digital safety systems should be paired with formal accountability and team-level support if motivational readiness is to translate into proactive safety behavior.

## 1 Introduction

A common assumption in safety governance research is that managerial attention to safety, once credibly signaled, will correspond to frontline safety outcomes in a relatively straightforward manner (Zohar, 2002; Clarke, 2013). Yet this linear assumption sits uncomfortably with a well-documented pattern: organizations whose leaders demonstrably prioritize safety still exhibit marked variation in frontline safety behavior (Christian et al., 2009), and safety motivation does not always convert into safety action (Bagozzi, 1992; Parker et al., 2010). Why does the same managerial priority produce different employee responses? Why does willingness to act safely not guarantee costly safety action? These questions are fundamentally psychological—they concern how individuals process organizational cues, form motivational states, and overcome interpersonal barriers to action.

To address these questions, this study integrates upper echelons theory with organizational psychology perspectives on the attitude–behavior gap, role perception, cognitive load, and psychological safety, using multi-source, time-lagged, multilevel data from 183 firms and 667 teams. Whereas much of the safety-management literature has emphasized structural and regulatory aspects of workplace safety, this study argues that the cross-level translation gap cannot be understood without examining the psychological processes through which organizational structures shape motivation differently from behavior. The contribution of the article is therefore not to compare governance tools in the abstract, but to explain how different organizational mechanisms alter the psychological thresholds separating willingness from costly action.

A central premise of this study is that frontline willingness and frontline action should be read as distinct manifestations of a broader organizational safety condition rather than as the simple sum of individual attitudes. Employees may report that safety matters while the organization still depends on inspections, reminders, or external pressure. What distinguishes deeper internalization is whether safety continues to organize decisions when production demands intensify and employees must act without direct supervision.

This perspective also clarifies why a signaling account, on its own, is incomplete. Leadership-signaling studies show that managerial cues affect employee perceptions and behavior (Zohar, 2002; Clarke, 2013), but signaling alone does not explain why organizations exposed to similar regulation and similar rhetoric still differ sharply in proactive safety action. The unresolved issue lies in the organizational pathway between managerial prioritization and frontline enactment.

This study addresses three linked questions: whether managerial safety cognition predicts the formation of heterogeneous organizational mechanisms, whether those mechanisms bridge the path to frontline willingness and action, and whether performance pressure activates or erodes that translation. These questions matter because they move the analysis from whether safety matters to how safety priorities become durable enough to survive organizational friction.

This study brings governance-oriented safety research into dialogue with organizational psychology by showing how organizational mechanisms shape the thresholds separating willingness from costly action.

Two specific gaps limit current understanding. First, most studies treat safety leadership or safety commitment as the proximal driver of frontline outcomes, implicitly adopting a signaling logic in which leaders transmit cues and employees respond (Zohar, 2002; Clarke, 2013; Jiang et al., 2024). Although these investigations have enriched our understanding of frontline safety behavior antecedents, they embed a strong linear presumption—that sufficient top-management signals will automatically produce frontline proactive safety practice. This framing identifies that managerial signals matter, but not how cognition becomes embedded in organizational structures that persist beyond individual signaling episodes. Second, although safety motivation and safety participation are routinely modeled as parallel outcome variables, few studies theorize why organizational mechanisms might relate to managerial cognition more effectively for one than the other. This omission is consequential: if the mechanisms that sustain motivational readiness differ from those that underwrite costly behavioral action, then a one-size-fits-all governance approach may systematically overestimate the reach of certain organizational investments—particularly technological ones.

Drawing on upper echelons theory (Hambrick and Mason, 1984; Hambrick, 2007) and the attention-based view (Ocasio, 1997; Joseph et al., 2024), this study proposes that managerial safety cognition is associated with frontline safety outcomes not through direct signaling, but through three organizational mechanisms: institutionalization, technological affordance, and team safety climate. This study further introduces performance pressure as a curvilinear boundary condition, drawing on the job demands–resources (JD-R) model (Bakker and Demerouti, 2007).

This study makes two contributions. It shifts the analytical starting point from observable leadership behavior to managerial attentional prioritization under competing demands, thereby proposing and initially testing a cross-level path from organizational-level cognitive origins to frontline outcomes. It also provides initial evidence that the organizational translation of managerial cognition is both mechanism-specific and outcome-dependent: technological affordance is associated with safety motivation but not with safety participation, whereas institutionalization and team safety climate are associated with both outcomes. Both contributions should be read as support for an explanatory framework rather than as proof of fully confirmed causal mechanisms.

## 2 Theoretical background and hypotheses

### 2.1 Managerial safety cognition as a cross-level cognitive antecedent

This study defines managerial safety cognition as the stable prioritization of safety within a manager’s attention structure when safety competes with operational goals such as profitability, cost containment, and delivery schedules. This construct is grounded in upper echelons theory, which holds that organizational outcomes reflect the cognitive orientations of top managers (Hambrick and Mason, 1984; Hambrick, 2007), and in the attention-based view, which emphasizes that what managers attend to determines how organizational resources are allocated (Ocasio, 1997; Joseph et al., 2024). Recent extensions of the attention-based view further emphasize that managerial attention is situated in material, social, temporal, and framing contexts (Brielmaier and Friesl, 2023), reinforcing the view that safety cognition reflects a contextually embedded attentional orientation rather than a purely dispositional attitude.

It is important to distinguish managerial safety cognition from two related constructs: safety commitment and top-management safety climate. This study proposes a three-level conceptual distinction.

#### 2.1.1 Definition boundary

Managerial safety cognition refers to a manager’s internal attentional prioritization and resource allocation preferences regarding safety issues—the cognitive upstream that determines which organizational domains receive sustained investment. Safety commitment, by contrast, typically refers to managers’ overt behavioral demonstrations of safety priority and their public signaling of safety values (Zohar, 2002; Clarke, 2013). Top-management safety climate refers to employees’ shared perceptions of organizational safety priority (Zohar and Luria, 2005).

#### 2.1.2 Causal positioning

In our framework, cognition functions as the upstream antecedent: it shapes which organizational mechanisms receive investment. Commitment functions as one mode of transmission—the behavioral signaling through which cognitive priorities become visible to employees. Climate is a downstream shared perception—the collective result of organizational members interpreting managerial signals and structural arrangements.

#### 2.1.3 Empirical status in this study

This study uses managerial safety cognition as an attentional-prioritization construct that foregrounds upstream resource allocation and agenda setting under competing demands. The Fruhen et al. (2014, 2019) items used here primarily capture internal prioritization and resource allocation rather than public behavioral signaling. Items such as allocating financial resources to safety or making safety a standing topic in strategic meetings describe upstream agenda-setting decisions rather than visible symbolic displays. This positioning complements commitment-based accounts by shifting the analytical focus from visible safety signaling to the cognitive ordering of safety within managerial attention structures.

This perspective should also be distinguished from adjacent constructs in the safety literature. Safety culture and climate describe shared priorities, safety management systems describe formal routines, and safety performance describes observable outcomes (Griffin and Neal, 2000; Fernández-Muñiz et al., 2007; Zohar and Luria, 2005). The present study instead focuses on the process through which externally mandated safety responsibility becomes more internally sustained.

This is why attentional prioritization is theoretically useful even if it overlaps empirically with commitment-related constructs. In organizations facing simultaneous demands, attention is scarce, and what becomes agenda-relevant is what is more likely to receive stable resources, managerial monitoring, and implementation follow-through (Ocasio, 1997; Hambrick, 2007). A cognition-centered formulation therefore directs analysis to the upstream ordering of problems inside managerial sensemaking, not simply to the downstream visibility of managerial rhetoric. That shift does not deny the importance of signaling; rather, it suggests that signaling is only one transmission channel through which a deeper attentional hierarchy becomes visible to the organization.

The argument also implies that cross-level translation is inherently fragile. Strategic intent is formulated at the top of the hierarchy, but frontline action is enacted amid local ambiguity, multiple accountabilities, and ongoing trade-offs. Unless safety prioritization becomes sedimented into organizational arrangements that survive beyond episodic exhortation, its downstream influence will weaken across levels. In this sense, the theoretical problem addressed here is not simply whether cognition matters, but how cognition becomes durable enough to shape frontline thresholds for motivation and action in the presence of hierarchical distance, local sensemaking, and competing operational demands.

To clarify the conceptual position of managerial safety cognition relative to two adjacent constructs that have received the most empirical attention in the safety leadership literature, Table 1 summarizes the distinctions across five dimensions identified as critical for construct demarcation: cognitive nature, behavioral manifestation, respondent source, theoretical position in the causal chain, and temporal stability. The distinctions below are analytical rather than empirical, because only managerial safety cognition was directly measured in the present study.

**Table 1:** Distinguishing managerial safety cognition from safety commitment and top-management safety climate.

Dimension	Managerial safety cognition (MC)	Safety commitment	Top-management safety climate
Cognitive nature	Internal attentional prioritization of safety relative to competing demands; an upstream allocation of scarce managerial attention to the safety domain	Value-based dedication to safety as a goal; a felt obligation toward safety expressed through the manager’s stance and intent	Employees’ shared cognitive perception of the relative priority that top management places on safety
Behavioral manifestation	Expressed indirectly through resource-allocation and agenda-setting decisions (e.g., budgeting, strategic-meeting placement, capital review, staffing priorities)	Expressed directly through visible managerial behaviors, such as signaling, modeling, public statements, and personal involvement in safety actions	Not itself a managerial behavior; rather, it reflects employees’ shared interpretation of managerial cues and structural arrangements
Respondent source	Senior managers (self-rated upstream cognition); the present study uses Fruhen et al. (2014, 2019) at T1	Typically senior managers (self-rated commitment) or, in observer designs, direct reports (Zohar, 2002; Clarke, 2013)	Frontline employees (perceiver-aggregated to the team or firm level; Zohar and Luria, 2005)
Theoretical position in causal chain	Upstream antecedent; shapes which organizational mechanisms receive sustained investment	Mid-stream transmission channel; one route through which underlying cognitive priorities become visible to organizational members	Downstream perceived outcome; reflects the collective interpretation of cognitive priorities and observable signals
Temporal stability	Conceptually expected to be relatively stable, given its grounding in managerial attentional structure under competing demands	Likely to be more episodic in visible expression, because commitment can be emphasized or de-emphasized across actions and occasions	Potentially more revisable, because it is updated through employees’ ongoing interpretation of managerial cues and organizational arrangements

The comparison is conceptual rather than empirical. The present study directly measured managerial safety cognition but did not simultaneously administer a separate safety-commitment scale.

### 2.2 Safety motivation and safety participation as distinct outcomes

Following the classic deconstruction of safety performance (Griffin and Neal, 2000), this study distinguishes two frontline outcomes: safety motivation (the willingness to engage in safety activities without external monitoring) and safety participation (actual proactive behaviors such as hazard reporting, peer intervention, and work stoppage). This distinction is grounded in the attitude–behavior gap literature (Bagozzi, 1992; Parker et al., 2010), which suggests that cognitive and attitudinal antecedents may not correspond symmetrically to willingness versus costly action.

However, cognitive prioritization does not automatically correspond to frontline outcomes. Between top-level strategic intent and frontline action, information attenuation occurs across organizational levels (Gioia and Chittipeddi, 1991; Maitlis and Christianson, 2014). The attention-based view explicitly recognizes that organizational attention is channeled through structural mechanisms that determine which issues receive sustained focus at lower levels (Ocasio, 1997). Therefore, the association between managerial cognition and frontline outcomes may require specific organizational mechanisms to bridge the cross-level gap. In this study, organizational translation refers to the process by which top-level safety prioritization is embedded into formal, technological, and social structures that shape frontline cognition and action.

Treating motivation and participation as distinct manifestations is especially important in high-risk contexts. Motivation indicates cognitive and normative legitimacy; participation indicates that the organization has also reduced the costs of acting.

Seen from this angle, the present study also extends attitude–behavior-gap research. The gap is often treated as an individual self-regulation problem: people endorse one thing but do another. Here, this study asks how organizational arrangements differentially lower the thresholds for willingness versus action. The implication is that the gap is not merely psychological in the narrow intrapersonal sense; it is also organizationally produced and organizationally maintained. Different embedding mechanisms may reduce informational uncertainty, role ambiguity, or interpersonal risk to different degrees, and it is precisely these differences that generate the asymmetric patterns examined in the results.

### 2.3 Organizational translation mechanisms

This study proposes three organizational mechanisms: institutionalization as a distal formal mechanism, technological affordance as a mid-range material mechanism, and team safety climate as a proximal relational mechanism. These mechanisms are complementary rather than mutually exclusive; they may co-occur and reinforce one another, but they are expected to differ in the strength and nature of their associations with motivational versus behavioral outcomes.

The choice of these three mechanisms is not arbitrary. Together, they capture three broad forms through which managerial priorities become organizationally embedded. Institutionalization reflects normative and procedural embedding: safety is coded into formal rules, accountability arrangements, and review loops, consistent with classical institutional theory (Selznick, 1957; Meyer and Rowan, 1977). Technological affordance reflects material embedding: safety priorities become inscribed into devices, platforms, monitoring systems, and digital reporting architectures (Gibson, 1979; Leonardi, 2011). Team safety climate reflects relational embedding: safety is interpreted and reproduced through shared expectations, interactional support, and local sensemaking (Zohar and Luria, 2005; Schneider et al., 2013). Together, they span formal, material, and relational channels of organizational translation.

Within this classification, middle managers are not treated as a separate fourth mechanism because they are implicated in all three translation channels in practice—implementing procedures, coordinating technological deployment, and shaping team-level interactions. They matter because they animate these carriers, but the explanatory focus remains on the structural carriers themselves.

A similar logic applies to other organizational mechanisms in the safety literature, including safety training, incentive systems, disciplinary arrangements, and lateral safety communication. These are content carried within the three structural channels rather than parallel channels of their own: training and disciplinary arrangements operate through formal procedural rules and audit cycles, placing them within the institutionalization channel; incentive systems are similarly embedded in formal accountability structures; and lateral safety communication operates through team-level normative dynamics, placing it within the team safety climate channel. The framework is not intended to enumerate every mechanism that can carry safety prioritization downstream.

The three mechanisms also differ in temporal durability. Institutionalization is comparatively slow to build but durable once embedded; technological affordance can be deployed more rapidly but depends on actual use and worker appropriation; team safety climate can shift more quickly but remains vulnerable to local supervisory and peer dynamics. These differences mean the mechanisms cannot be treated as functionally equivalent organizational resources.

This heterogeneity is what makes a cross-level embedding perspective useful: formal, material, and relational channels transform the same upstream priority into different downstream affordances. Some make hazards more visible, some make action more legitimate, and some make intervention more socially tolerable. This difference in functional reach is central to the asymmetry argument tested in the results.

The three mechanisms can also be read as a translation chain: formal institutions define expectations, technological infrastructures make risks more visible, and relational climates shape whether acting on that information is socially viable.

**Institutionalization**. When managers prioritize safety, they may encode this priority into formal rules, accountability structures, and procedural safeguards (Fernández-Muñiz et al., 2007; Naveh et al., 2005). Drawing on role theory (Morrison, 1994), formal institutions clarify that safety-related activities are expected in-role behaviors, reducing role ambiguity and establishing clear responsibility boundaries.

*H1a:* Managerial safety cognition is positively associated with institutionalization.

**Technological affordance**. Managers who prioritize safety may invest in digital systems that support risk monitoring and safety decision-making (Leonardi, 2011; Cagno et al., 2024). Drawing on cognitive load theory (Sweller, 1988), technology may reduce the extraneous cognitive load associated with risk identification by transforming hidden hazards into visible cues.

*H1b:* Managerial safety cognition is positively associated with technological affordance.

**Team safety climate**. A manager’s safety prioritization may also be reflected in resource allocation decisions that signal safety’s importance (Zohar, 2000). Through social information processing (Salancik and Pfeffer, 1978), these signals are collectively interpreted and crystallized into shared team perceptions of safety priority (Zohar and Luria, 2005).

*H1c:* Managerial safety cognition is positively associated with team safety climate.

### 2.4 Asymmetric associations: motivation versus behavior

This study expects the three mechanisms to differ in their association with the motivational versus behavioral dimensions. This asymmetry may arise because the motivational dimension primarily requires cognitive accessibility and attitudinal reorientation, whereas the behavioral dimension additionally requires overcoming interpersonal costs—social anxiety, role conflict, and relational tension. This distinction parallels recent voice and silence research (Morrison, 2023), which treats speaking up and remaining silent as psychologically distinct responses to perceived risk rather than mirror-image outcomes.

The asymmetry argument can be restated as a dual-threshold problem. To awaken motivation, organizations must make safety cognitively accessible, normatively legitimate, and sufficiently worthwhile to merit attention. To support participation, organizations must do all of that and also reduce the anticipated costs of acting—especially the interpersonal, political, and role-related costs attached to interrupting work, challenging coworkers, or escalating concerns upward. For this reason, the same mechanism may plausibly perform well at the first threshold yet stall at the second.

Institutionalization operates through formal accountability structures that establish safety-related activities as normatively expected role behaviors (Morrison, 1994), potentially facilitating both motivational engagement and behavioral follow-through:

Institutionalization is expected to matter across both thresholds because it reduces ambiguity about what counts as appropriate conduct and who bears responsibility for acting. Formalization helps convert abstract safety priorities into role-prescribed expectations, thereby lowering uncertainty about whether speaking up, reporting, or intervening is legitimate. In motivational terms, this may reinforce the sense that safety-related effort is part of how one is expected to work. In behavioral terms, it provides procedural cover for actions that might otherwise appear disruptive or overreaching. This dual relevance explains why institutionalization is theorized as a bridge not only to willingness but also to enactment.

*H2a:* Institutionalization is positively associated with safety motivation.

*H3a:* Institutionalization is positively associated with safety participation.

Technological affordance primarily operates by reducing the extraneous cognitive load of risk identification (Sweller, 1988) and strengthening safety self-efficacy (Bandura, 1997). However, technology may be less capable of reducing the interpersonal costs of costly safety behaviors such as work stoppage or peer confrontation (Detert and Edmondson, 2011). Empirical evidence from offshore high-hazard contexts is consistent with this asymmetry, showing that hazard recognition alone is insufficient for safety voice, which additionally requires supervisory support and job control (Mathisen et al., 2022). This study therefore expects technological affordance to be more strongly associated with motivational readiness than with behavioral implementation. For directional completeness and consistency with prior enabling-mechanism research, this study retains a baseline positive prediction for both outcomes; however, the sharper theoretical implication is the relative-difference claim that any behavioral association should be weaker than the corresponding motivational association:

*H2b:* Technological affordance is positively associated with safety motivation.

*H3b:* Technological affordance will show a weaker association with safety participation than with safety motivation.

*Analytical expectation*. Given the asymmetric pathway argued above, this study further expects that technological affordance may exhibit no statistically significant direct association with safety participation when evaluated alongside the corresponding association with safety motivation. This study frames this expectation as analytical rather than as a formal hypothesis, because a nonsignificant direct association is an evidentiary expectation rather than a directional effect hypothesis.

Team safety climate provides proximal relational context. Drawing on psychological safety theory (Edmondson, 1999), positive climate may reduce the social anxiety associated with challenging safety behaviors, providing relational trust and normative backing:

Team safety climate should also reach both thresholds, but through a different mechanism. Whereas institutionalization works through formal authorization, climate operates through local expectations and relational reassurance. A positive climate indicates that safety concerns can be voiced without social sanction, that coworkers and supervisors are likely to treat such conduct as legitimate, and that challenging unsafe routines will not automatically trigger blame or exclusion. This relational support is especially important for high-cost proactive behavior. Motivation may be fostered by many kinds of organizational encouragement, but costly action often requires a social environment in which employees believe that the interpersonal consequences of acting will be manageable. This pattern is consistent with recent psychological-safety reviews characterizing psychological safety as a state of reduced interpersonal risk that is especially relevant for high-cost participation (Edmondson and Bransby, 2023), and with safety-voice evidence that different supervisory styles produce differentiated patterns of upward safety communication (Curcuruto and Griffin, 2023).

*H2c:* Team safety climate is positively associated with safety motivation.

*H3c:* Team safety climate is positively associated with safety participation.

### 2.5 Mediation

If managerial safety cognition is associated with frontline outcomes primarily through organizational mechanisms rather than direct signaling, the direct cognitive–outcome association should attenuate when mechanisms are included. Because institutionalization and technological affordance are modeled at the firm level whereas team safety climate originates at the team level and is decomposed into between- and within-firm components, the three mediators do not all reside at the same organizational level. The mediation architecture therefore combines two pathway types: firm-level 2-2-1 pathways, whose mediators are institutionalization or technological affordance, and a 2-1-1 cross-level pathway centered on team safety climate (The digits follow standard multilevel-mediation notation, applied in the order predictor–mediator–outcome, with “2” denoting a firm-level variable and “1” denoting a team-level variable).

The mediation logic follows directly from the theoretical shift from signaling to embedding. If managerial safety cognition works primarily by shaping where organizational attention and resources are invested, then its downstream relevance should be visible in the organizational carriers that translate priorities across levels. This expectation is stronger than a simple “leaders matter” claim but weaker than a strict causal chain. The article therefore predicts attenuation of the direct cognition–outcome association once the three mechanisms are entered, while recognizing that the observed indirect paths are most appropriately interpreted as multilevel association patterns consistent with organizational mediation. This mediation logic is grounded in the attention-based view that managerial attention becomes consequential through organizational structures that channel attention and sensemaking across levels (Ocasio, 1997; Gioia and Chittipeddi, 1991; Maitlis and Christianson, 2014).

The distinction between 2-2-1 and 2-1-1 pathways is consequential here. Institutionalization and technological affordance are firm-level properties rated by organizational informants, so their indirect associations with team outcomes take a 2-2-1 form. Team safety climate is generated from employee ratings and then decomposed into between- and within-firm components, so the climate pathway contains a 2-1-1 cross-level element. Making this structure explicit clarifies that the article does not assume all mechanisms operate at the same level or in the same way. Instead, the analysis deliberately preserves level-specific heterogeneity in the translation process.

*H4a:* Institutionalization mediates the association between managerial safety cognition and frontline safety outcomes.

*H4b:* Technological affordance mediates the association between managerial safety cognition and frontline safety outcomes.

*H4c:* Team safety climate mediates the association between managerial safety cognition and frontline safety outcomes.

A note on inferred mechanisms. The asymmetry argument advanced in Section 2.4 distinguishes between mechanisms that primarily increase the cognitive accessibility of safety or reduce informational thresholds, and mechanisms that additionally reduce the interpersonal costs of acting on safety concerns. Interpersonal cost, role conflict, social anxiety, and psychological safety risk are treated as theoretically inferred psychological costs within this framework. The present analysis tests whether organizational carriers exhibit the expected asymmetric pattern across motivation and participation; direct measurement of these inferred psychological costs would be a valuable next step for future research.

### 2.6 Performance pressure as a curvilinear boundary condition

Drawing on the JD-R model (Bakker and Demerouti, 2007; Bakker et al., 2023), the challenge–hindrance stressor framework (Cavanaugh et al., 2000; LePine et al., 2005), and activation-theory reasoning, this study proposes that moderate performance pressure may function as an activating challenge demand that focuses accountability and energizes role execution, whereas excessive pressure may become a hindrance demand that depletes cognitive and regulatory resources (Hobfoll, 1989). More recent integrative work on the challenge–hindrance stressor framework reaffirms this duality, treating pressure as carrying both activating and depleting functions whose balance depends on appraisal and available resources (LePine, 2022). Recent systematic evidence further indicates that the production pressure–safety relationship is heterogeneous rather than uniformly negative (Hashemian and Triantis, 2023):

Performance pressure is introduced not as a rival theory of safety behavior, but as a stringent contextual test of whether organizational embedding is robust under resource tension. A system that only supports safety under low-demand conditions may reflect procedural compliance rather than genuine internalization. By contrast, an organization with internally sustained safety strength should display some capacity to maintain—or even temporarily sharpen—the behavioral relevance of its safety arrangements under moderate pressure. This is why pressure is analytically valuable in the present model: it probes whether the mechanisms linking managerial cognition to frontline action are fragile, activated, or depleted when operational demands intensify.

The prediction of an inverted-U relationship follows because moderate pressure can focus attention, increase the salience of accountability, and prompt fuller use of existing routines and tools, whereas excessive pressure can crowd out reflective capacity, erode rule adherence, and normalize short cuts (Cavanaugh et al., 2000; LePine et al., 2005). In this sense, pressure is not assumed to be uniformly beneficial or uniformly harmful. Its function depends on intensity. Under moderate levels, it may operate as an activating challenge demand; beyond a threshold, it becomes a hindrance demand that depletes the very cognitive and regulatory resources required for proactive safety action.

This study focuses the moderation hypotheses on institutionalization and technological affordance because both represent firm-level organizational mechanisms whose behavioral relevance may vary with firm-level performance pressure. Team safety climate, by contrast, is modeled as a team-derived relational mechanism and decomposed into between- and within-firm components. Extending the same firm-level curvilinear pressure logic to this decomposed climate pathway would introduce a different cross-level moderation structure, which is beyond the scope of the present analysis.

*H5a:* Performance pressure exhibits an inverted-U-shaped moderating effect on the association between institutionalization and safety participation.

*H5b:* Performance pressure exhibits an inverted-U-shaped moderating effect on the association between technological affordance and safety participation.

**Figure 1:**
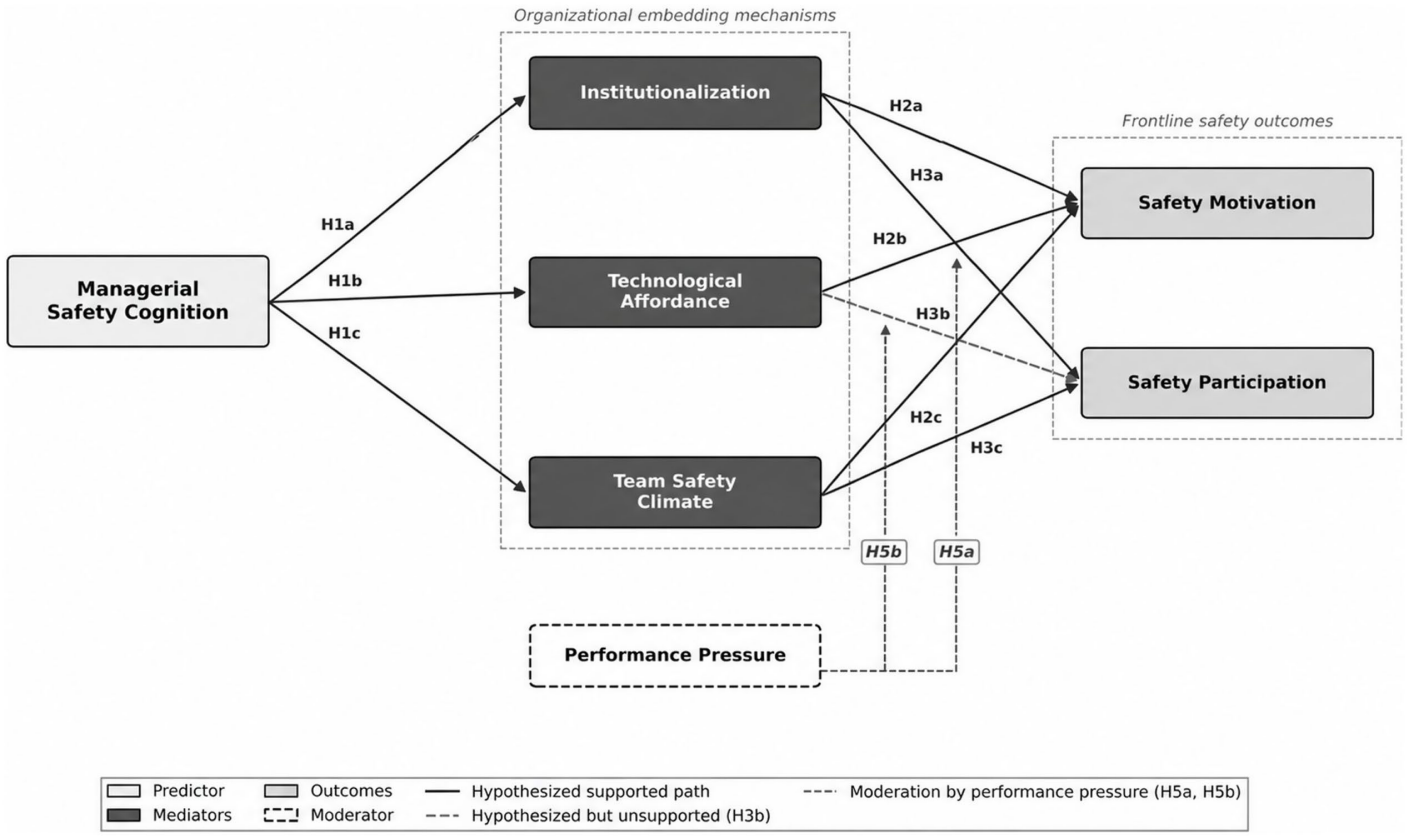
Conceptual model and hypothesized relationships. Solid arrows denote hypothesized positive paths. The dashed pathway (H3b) denotes the theoretically weaker technological-affordance-to-safety-participation link.

The conceptual model is presented in Figure 1.

## 3 Materials and methods

### 3.1 Sample and procedure

Data were collected from firms in China across high-risk industries, including manufacturing (25.7%, *n* = 47), hazardous chemicals (16.4%, *n* = 30), energy and power (10.9%, *n* = 20), non-coal mining (10.4%, *n* = 19), construction (9.3%, *n* = 17), coal mining (9.3%, *n* = 17), transportation (6.0%, *n* = 11), and other sectors (12.0%, *n* = 22). Participating firms were recruited through industry associations and safety regulatory agencies across multiple provinces. Firms ranged from small enterprises with fewer than 100 employees to large organizations with more than 1,000 employees and included state-owned (38%), privately owned (47%), and foreign-invested enterprises (15%); the employee sample was predominantly male (approximately 78%), with an average organizational tenure of approximately 7 years. Frontline respondents were employees engaged in production, construction, mining, hazardous-chemical, maintenance, transportation, energy/power, or other safety-critical operational work.

The study employed a three-wave, multi-source nested matched survey with a three-month lag between adjacent waves. At T1, senior managers rated firm characteristics, performance pressure, and managerial safety cognition. At T2, safety-function leaders rated institutionalization and technological affordance, while frontline employees rated team safety climate. At T3, frontline employees rated safety motivation and safety participation, and direct supervisors provided independent behavioral ratings for cross-source validation. The auditable field process began with 312 firms at T1; 237 firms remained matched through T2, yielding 892 teams at that stage. After invalid questionnaires, cases with missing key information, and records that could not be matched across waves or informant sources were removed, the final analytic sample consisted of 183 firms and 667 teams. A detailed sample-flow summary is reported in Supplementary Table S8.

Participation was voluntary and based on informed consent. Participants were informed of the academic purpose of the study, the survey procedure, and the confidentiality protections before completing the questionnaires. Responses were collected without names or contact information. Non-identifying numeric identifiers were used only to support the nested data structure and cross-wave or cross-source matching and contained no personally identifying information. Within the final retained analytic sample, T2 included 11,691 valid frontline-employee responses for team safety climate, and T3 included 12,379 valid frontline-employee responses for safety motivation and safety participation. The final sample averaged 3.64 teams per firm. The employee-response counts correspond to average effective team response sizes of 17.52 for team safety climate and 18.56 for safety motivation/participation. The supervisor-rated behavioral validation subsample included 366 teams, representing 55% of the total team sample, and was drawn from all 183 firms.

### 3.2 Measures

All core constructs were measured using established scales adapted for the Chinese organizational safety context. Items were translated from English to Chinese and back-translated by independent bilingual researchers following the back-translation guidelines of Brislin (1986). A pilot study with 45 safety managers and 120 frontline workers confirmed item clarity and cultural appropriateness. Complete item wordings are provided in Supplementary Appendix A. Unless otherwise noted, all subjective items used 5-point Likert scales.

The measurement strategy was designed to align construct level with informant competence. Managerial safety cognition and performance pressure were rated by senior managers because these constructs concern strategic prioritization under competing organizational goals. Institutionalization and technological affordance were rated by safety-function leaders because they possess the most comprehensive knowledge of organizational procedures, digital systems, and compliance loops. Team safety climate, safety motivation, and safety participation were generated from frontline employee ratings because these constructs concern shared local interpretation and enacted behavior in day-to-day work. This alignment does not eliminate all measurement concerns, but it reduces the mismatch that would arise if structurally distant respondents were asked to evaluate constructs outside their zone of practical knowledge.

A second measurement principle is equally important: the present article does not treat frontline motivation and participation as isolated team-level outcomes in the ordinary sense. They are used as observable micro-manifestations through which a broader organizational condition becomes empirically identifiable. This means that the analysis is not merely asking whether one team behaves more safely than another. It is asking whether firms differ systematically in their ability to generate widespread motivational readiness and stable proactive enactment across constituent teams. That distinction helps justify the multilevel aggregation strategy and explains why between-firm variance in the frontline constructs is theoretically central rather than incidental.

#### 3.2.1 Independent variable

**Managerial safety cognition**. The Fruhen et al. (2014, 2019) scale, completed by senior managers at T1, containing 8 items (*α* = 0.890).

#### 3.2.2 Moderating variable

**Performance pressure**. Adapted from Bakker and Demerouti (2007), completed by senior managers at T1, containing 4 items (*α* = 0.849).

#### 3.2.3 Mediating variables

**Institutionalization**. Based on Naveh et al. (2005), completed by safety-function leaders at T2, containing 6 items (*α* = 0.902).

**Technological affordance**. Based on Leonardi (2011), completed by safety-function leaders at T2, containing 6 items (*α* = 0.908).

A methodological note is warranted because institutionalization and technological affordance were both rated by safety-function leaders (Kumar et al., 1993). This improves informant fit but does not eliminate the possibility of same-source inflation between the two mechanism measures. To limit that risk, both variables were entered simultaneously in the multilevel models, and cross-source checks were conducted with supervisor-rated behavior. The design therefore reduces—but does not fully eliminate—the possibility that the core asymmetry is a same-source artifact.

**Team safety climate**. Adapted from Zohar and Luria (2005), completed by frontline employees at T2, containing 8 items (*α* = 0.967).

#### 3.2.4 Dependent variables

**Safety motivation (SM)**. Adapted from Griffin and Neal (2000), completed by frontline employees at T3, containing 4 items (*α* = 0.938).

**Safety participation (SP)**. Adapted from Neal and Griffin (2006), completed by frontline employees at T3, containing 8 items (*α* = 0.969).

#### 3.2.5 Control variables

At the firm level, models controlled for firm background characteristics from the T1 executive survey (A_CTRL1–A_CTRL10), including industry sector, ownership type, firm age, workforce size, revenue category, risk level, group-affiliation status, recent accident history, safety-investment ratio, and baseline digitalization level. In addition, four factual safety-condition controls recorded by the safety department were included as conservative contextual covariates: B_FACT1, whether a near-miss incident log was established (binary); B_FACT2, whether a management-of-change system was established (binary); B_FACT3, whether high-risk operation approval left a documented trail (binary); and B_FACT4, the number of safety-system revisions in the past 12 months (count). These four factual controls were entered as separate standardized covariates rather than as summary PCA components. Their definitions, coding, and provenance are documented in Supplementary Note 1, and the complete coefficient tables in Supplementary Tables S1–S4 retain the corresponding A_CTRL1_z–A_CTRL10_z and B_FACT1_z–B_FACT4_z entries.

### 3.3 Data aggregation

Team safety climate, safety motivation, and safety participation were generated from individual employee ratings and aggregated to the team level. Mean within-group agreement indices (*r_wg_*) were 0.716, 0.729, and 0.704, with corresponding medians of 0.720, 0.738, and 0.715, respectively. Team-level *r_wg_* values ranged from 0.190 to 0.920 for team safety climate, 0.325 to 0.973 for safety motivation, and 0.328 to 0.906 for safety participation. Some teams fell below the conventional 0.70 threshold (38.8, 31.5, and 42.4%, respectively), which may reflect within-team heterogeneity in high-risk operational settings as well as measurement variation. To assess robustness, this study re-estimated the core mechanism regressions using a strict subsample retaining only teams with *r_wg_* ≥ 0.70 across all three frontline constructs (*N* = 191 teams nested within 122 firms). The asymmetric pattern of mechanism associations replicated in this strict subsample; results are reported in Supplementary Table S9. In the restricted high-agreement subsample (191 teams from 122 firms), the central technological asymmetry was retained: technological affordance was associated with safety motivation (*β* = 0.245, *p* < 0.05) but not with safety participation (*β* = 0.115, n.s.). The institutional pathway also remained associated with both outcomes, including safety motivation (*β* = 0.353, *p* < 0.01) and safety participation (*β* = 0.356, *p* < 0.01). The between-firm climate path showed an almost identical point estimate for safety motivation relative to the full sample (*β* = 0.135 vs. 0.136) but no longer reached conventional significance, plausibly reflecting reduced statistical power in the restricted subsample; its association with safety participation remained significant (*β* = 0.299, *p* < 0.01). Intraclass correlation coefficients for the full sample were ICC(1) = 0.188, 0.192, and 0.201, and ICC(2) = 0.802, 0.816, and 0.823, respectively.

Table 2 reports the interrater agreement (*r_wg_*) and intraclass correlation (ICC) statistics supporting aggregation for the three team-level variables.

**Table 2:** Data aggregation validity for team-level variables.

Construct	Avg. team size	Mean *r_wg_*	ICC(1)	ICC(2)
Team safety climate	17.52	0.716	0.188	0.802
Safety motivation	18.56	0.729	0.192	0.816
Safety participation	18.56	0.704	0.201	0.823

Safety motivation and safety participation were aggregated to the team level rather than modeled at the individual level because the theoretical focus is on between-firm systematic differences in the “widespread awakening” of motivation and the “stable consolidation” of behavior, with within-firm team variance treated as localized implementation heterogeneity. This aggregation is consistent with the cross-level measurement paradigm in which macro-level latent states are identified through micro-level observable indicators.

The aggregation logic therefore serves both a statistical and a theoretical purpose. Statistically, *r_wg_* and ICC values establish that frontline perceptions and behaviors contain enough within-team convergence and between-team differentiation to support aggregation. Theoretically, aggregation allows the article to identify how macro-level safety prioritization becomes visible in repeated local manifestations without assuming that every team inside a firm is perfectly homogeneous. Within-firm variance is not treated as noise to be discarded; it is treated as implementation heterogeneity nested within a broader firm-level translation process.

### 3.4 Analytical strategy

This study adopted a segmented multilevel modeling strategy. Firm-level antecedent paths from managerial safety cognition to institutionalization, technological affordance, and the between-firm component of team safety climate were estimated using OLS regression with heteroscedasticity-robust standard errors—appropriate because both predictor and outcome reside at the firm level. Cross-level outcome paths were estimated using two-level (team–firm) random-intercept multilevel linear models.

This study chose segmented HLM over simultaneous MSEM for two reasons: first, segmented estimation more precisely matches the two-stage theoretical logic in which macro-cognitive antecedents drive micro-mechanism penetration; second, the firm-level mechanisms are measured by single informants while team-level outcomes include dual assessment channels—forcing simultaneous estimation would amplify instability given the limited firm sample size (*N* = 183). To confirm robustness, this study conducted a supplementary random-intercept MSEM (see Section 4.7) and found that core asymmetric patterns remained consistent.

Team safety climate was decomposed into a between-firm mean (capturing between-firm shared component) and a within-firm group-mean-centered deviation (capturing within-firm heterogeneity). Firm-level continuous variables were grand-mean centered. For multilevel mediation, Monte Carlo parametric resampling (20,000 simulations) was used. Because the model includes both firm-level mediators (institutionalization and technological affordance) and a decomposed team-level climate pathway, the mediation tests include 2-2-1 and 2-1-1 cross-level components. This multilevel mediation architecture follows recent multilevel work treating team- or organization-level cues as antecedents shaping individual-level outcomes through psychological mechanisms, with cross-level moderation by team or organizational context (Li and Tang, 2022; Li and Hahn, 2026). For moderation, linear terms, quadratic terms, and their interactions with mechanism variables were introduced. The core estimation equations are shown below; full coefficient tables, including all control variables, are reported in Supplementary Tables S1–S4.

The distinction between aggregation statistics and null-model ICCs should also be emphasized. The *r_wg_*, ICC(1), and ICC(2) values reported for frontline constructs justify the upward aggregation of employee ratings to the team level. By contrast, the larger ICCs obtained from the unconditional multilevel models quantify the degree to which variation in the focal outcomes resides between firms rather than purely within firms. The two sets of statistics answer different questions: the first asks whether employees within teams are sufficiently aligned to permit aggregation; the second asks whether the nested data structure is strong enough to require multilevel estimation. Reporting both is important because the article treats frontline manifestations as team-generated but firm-differentiated outcomes.

Core model equations.

Level 1 (team within firm): $Y_{\mathrm{ij}}=\beta_{0j}+\beta_{1}({\mathrm{SC}}_{\text{within},\mathrm{ij}})+r_{\mathrm{ij}}$

Level 2 total-effect model: $\beta_{0j}=\gamma_{00}+\gamma_{01}{\mathrm{MC}}_{j}+\Sigma \gamma_{c}\;\text{Control}s_{j}+u_{0j}$

Level 2 mechanism model: $\begin{array}{l} \;\;\;\;\;\;\;\;\;\;\;\;\;\;\;\;\;\;\;\;\;\beta_{0j}=\gamma_{00}+\gamma_{01}{\mathrm{MC}}_{j}+\gamma_{02}{\mathrm{II}}_{j}+\gamma_{03}{\mathrm{TE}}_{j}+\gamma_{04}\\ \;\;\;\;\;\;\;\;\;\;\;\;\;\;\;\;\;\;\;\;\;\;\;\;\;\;\;\;\;\;\;\;\;{\mathrm{SC}}_{\text{between},j}+\Sigma \gamma_{c}\;\text{Control}s_{j}+u_{0j} \end{array}$

Firm-level antecedent models: ${\mathrm{II}}_{j}=\alpha_{0}+\alpha_{1}{\mathrm{MC}}_{j}+\;\Sigma \alpha_{c}\;\text{Control}s_{j}+\varepsilon_{j}$ ; ${\mathrm{TE}}_{j}=\alpha_{0}+\alpha_{1}{\mathrm{MC}}_{j}+\Sigma \alpha_{c}\;\text{Control}s_{j}+\varepsilon_{j}$ ; ${\mathrm{SC}}_{j}=\alpha_{0}+\alpha_{1}{\mathrm{MC}}_{j}+\Sigma \alpha_{c}\;\text{Control}s_{j}+\varepsilon_{j}$

Curvilinear moderation model: $\begin{array}{l} \beta_{0j}=\gamma_{00}+\gamma_{01}M_{j}+\gamma_{02}{\mathrm{PPc}}_{j}+\gamma_{03}{\mathrm{PPc}}_{j}^{2}+\gamma_{04}(M_{j}\times {\mathrm{PPc}}_{j})\\ +\gamma_{05}(M_{j}\times {\mathrm{PPc}}_{j}^{2})+\Sigma \gamma_{c}\;\text{Control}s_{j}+u_{0j} \end{array}$

In these equations, subscripts i and j index teams within firms and firms, respectively; $Y_{\mathrm{ij}}$ denotes the focal outcome (safety motivation or safety participation, estimated separately); ${\mathrm{PPc}}_{j}$ is performance pressure grand-mean centered, with ${\mathrm{PPc}}_{j}^{2}$ its quadratic term; γ,β,α are coefficients at Level 2, Level 1, and the firm-level OLS antecedent paths, respectively; and $r_{\mathrm{ij}},\;u_{0j},\;\varepsilon_{j}$ are the residual terms. $M_{j}$ , used in the curvilinear moderation model only, denotes either institutionalization or technological affordance.

## 4 Results

### 4.1 Measurement model validation

This study first evaluated the measurement structure of the focal constructs before estimating the structural paths. Table 3 reports the model-fit results.

**Table 3:** Measurement model fit indices.

Model	*χ^2^*	*df*	*χ^2^*/*df*	CFI	TLI	RMSEA
Firm-level four-factor CFA (MC, PP, II, TE)	270.984	246	1.102	0.989	0.988	0.024
Team climate single-factor MCFA (SC)	38.936	20	1.947	0.996	0.995	0.038
Motivation and behavior two-factor MCFA (SM, SP)	263.694	53	4.975	0.972	0.965	0.077
Seven-factor aggregated CFA (all)	2202.800	874	2.520	0.940	0.935	0.048

The firm-level CFA showed excellent fit for managerial safety cognition, performance pressure, institutionalization, and technological affordance, while the multilevel factor solutions for climate and the two frontline manifestations were acceptable for applied multilevel research.

Table 4 reports the construct reliability and discriminant validity statistics. Composite reliability values ranged from 0.850 to 0.969, AVE values ranged from 0.534 to 0.793, and all HTMT values between construct pairs fell below the conservative 0.85 threshold (Henseler et al., 2015).

**Table 4:** Construct reliability and discriminant validity.

Construct	CR	AVE	HTMT range
Managerial safety cognition (MC)	0.895	0.534	0.209–0.583
Performance pressure (PP)	0.868	0.627	0.015–0.583
Institutionalization (II)	0.907	0.621	0.255–0.560
Technological affordance (TE)	0.916	0.648	0.284–0.560
Team safety climate (SC)	0.969	0.793	0.209–0.430
Safety motivation (SM)	0.850	0.588	0.293–0.542
Safety participation (SP)	0.917	0.584	0.196–0.542

A particular concern arises from the firm-level correlation between safety motivation and safety participation reported in Table 5 (*r* = 0.829, *N* = 183), which could be interpreted as suggesting construct redundancy. Two checks indicate that this is not the case. First, the individual-level Pearson correlation between SM and SP scale scores was 0.635, with a disattenuated correlation of 0.666, far from unity. Second, the heterotrait–monotrait ratio for the SM–SP pair remained below the conservative 0.85 threshold at every level of aggregation: HTMT = 0.665 at the individual level (*N* = 12,379), 0.779 at the team level (*N* = 667), and 0.834 at the firm level (*N* = 183) (Henseler et al., 2015). Notably, even at firm aggregation—the level at which between-cluster correlation is most susceptible to upward inflation—the HTMT remained under threshold. The substantially higher firm-aggregated correlation in Table 5 is therefore consistent with an aggregation effect rather than latent-construct redundancy: averaging across many employees attenuates random measurement error and makes between-firm true-score covariance more visible (Bliese, 2000; Lüdtke et al., 2008). For the regressions in which the mechanism predictors were entered simultaneously, variance inflation factors ranged from 1.37 to 2.05, well below the conventional 5.0 threshold; full VIF values are reported in Supplementary Table S6. Additional item-level CFA-based discriminant-validity diagnostics, including Fornell–Larcker comparisons, are reported in Supplementary Table S7.

**Table 5:** Between-firm descriptive statistics and correlations (*N* = 183).

Variable	Mean	SD	1	2	3	4	5	6	7
1. SM	2.482	0.329	—						
2. SP	2.648	0.359	0.829***	—					
3. MC	3.547	0.602	0.367***	0.341***	—				
4. II	2.958	0.586	0.389***	0.389***	0.546***	—			
5. TE	2.714	0.582	0.423***	0.281***	0.562***	0.365***	—		
6. SC	2.713	0.326	0.347***	0.415***	0.510***	0.321***	0.370***	—	
7. PP	2.691	0.843	0.642***	0.583***	−0.006	0.035	0.072	0.066	—

Team-derived constructs (SM, SP, and SC) were first aggregated to the firm mean, and all coefficients are reported as between-firm descriptive correlations (*N* = 183) to avoid pseudo-replication and inflated degrees of freedom in cross-level bivariate reporting. Discriminant-validity evidence at individual and aggregation levels, including HTMT estimates, individual-level correlations, and item-level CFA-based Fornell–Larcker diagnostics, is reported in Section 4.1 and Supplementary Table S7. The higher firm-aggregated correlation between SM and SP (*r* = 0.829) should therefore be interpreted alongside the item-level evidence and is consistent with aggregation-based attenuation of random measurement error rather than latent-construct redundancy. *p < 0.05, **p < 0.01, ***p < 0.001. SM, Safety motivation; SP, Safety participation; MC, Managerial safety cognition; II, Institutionalization; TE, Technological affordance; SC, Team safety climate; PP, Performance pressure.

### 4.2 Descriptive statistics

Descriptive statistics and between-firm correlations are reported in Table 5.

Performance pressure showed near-zero correlation with managerial safety cognition but positive correlations with both outcomes. Institutionalization and technological affordance were positively correlated (*r* = 0.365); diagnostics indicated no problematic multicollinearity.

### 4.3 Cross-level main effects

Unconditional null models with teams nested within firms yielded firm-level ICCs of 0.596, 0.671, and 0.721 for team safety climate, safety motivation, and safety participation, respectively, confirming substantial between-firm variance and the necessity of multilevel modeling.

Table 6 reports the cross-level main-effect estimates and the organizational mechanism models for safety motivation and safety participation.

**Table 6:** Cross-level main effects and organizational mechanism tests.

Variable	SM (total)	SM (mechanism)	SP (total)	SP (mechanism)
MC	0.252*** (0.070)	0.061 (0.081)	0.239** (0.074)	0.028 (0.084)
II	—	0.234** (0.088)	—	0.296** (0.091)
TE	—	0.179* (0.081)	—	0.064 (0.084)
SC (between)	—	0.136* (0.065)	—	0.261*** (0.066)
SC (within)	—	−0.018 (0.023)	—	0.042* (0.020)

Robust standard errors in parentheses. *p < 0.05, **p < 0.01, ***p < 0.001. Complete coefficient sets for all control variables are reported in Supplementary Tables S1–S3.

MC was significantly positively associated with SM (*β* = 0.252, *p* < 0.001) and SP (*β* = 0.239, *p* < 0.01), indicating that top-level safety prioritization corresponds to frontline outcomes at the total-effect level. In firm-level antecedent models (OLS with robust standard errors), MC was significantly positively associated with II (*β* = 0.220, *p* < 0.001), TE (*β* = 0.437, *p* < 0.001), and SC (*β* = 0.451, *p* < 0.001), supporting H1a, H1b, and H1c, respectively.

When all three organizational mechanisms were entered simultaneously, a differentiated pattern emerged. Institutionalization (*β* = 0.234, *p* < 0.01), technological affordance (*β* = 0.179, *p* < 0.05), and the between-firm component of team safety climate (*β* = 0.136, *p* < 0.05) were all positively associated with safety motivation, supporting H2a, H2b, and H2c.

For safety participation, however, the pattern was more selective. Institutionalization (*β* = 0.296, *p* < 0.01) and the between-firm component of team safety climate (*β* = 0.261, *p* < 0.001) remained significant, supporting H3a and H3c. Technological affordance was not statistically significant (*β* = 0.064, *p* > 0.05), consistent with the analytical expectation stated in Section 2.4 that the direct association with safety participation may not reach conventional significance when evaluated alongside the corresponding association with safety motivation.

*Formal asymmetry test*. The coefficient-difference test directly evaluates the article’s central asymmetry claim. A Wald-type comparison supported H3b: the association of TE with SM was significantly stronger than its association with SP (Δ*β* = 0.115, *z* = 2.03, *p* = 0.042), indicating a statistically reliable difference in the magnitude of association across outcomes—not merely an artifact of one coefficient being significant and the other not. By contrast, the coefficients for II did not differ significantly across the two outcomes (Δ*β* = −0.062, *z* = −0.89, *p* = 0.370).

In addition, the within-firm component of team safety climate showed a small positive association with safety participation (*β* = 0.042, *p* < 0.05) but was not associated with safety motivation.

### 4.4 Mediation tests

This study then estimated path-specific indirect associations using Monte Carlo confidence intervals (Preacher and Selig, 2012). Table 7 reports the mediation results.

**Table 7:** Multilevel mediation tests (Monte Carlo 20,000 simulations).

Indirect path	Effect	95% CI
MC → II → SM	0.052	[0.014, 0.106]
MC → TE → SM	0.079	[0.006, 0.163]
MC → SC (between) → SM	0.052	[0.010, 0.107]
MC → II → SP (self)	0.066	[0.018, 0.130]
MC → TE → SP (self)	0.028	[−0.030, 0.092]
MC → SC (between) → SP (self)	0.099	[0.044, 0.172]

After entering all three mechanisms, the direct association between MC and both outcomes attenuated to non-significance. This pattern is consistent with organizational mediation in a time-lagged multilevel design, while the observational nature of the data warrants causal caution.

Institutionalization and the between-firm component of team safety climate each showed significant indirect associations with both safety motivation and safety participation.

**Figure 2:**
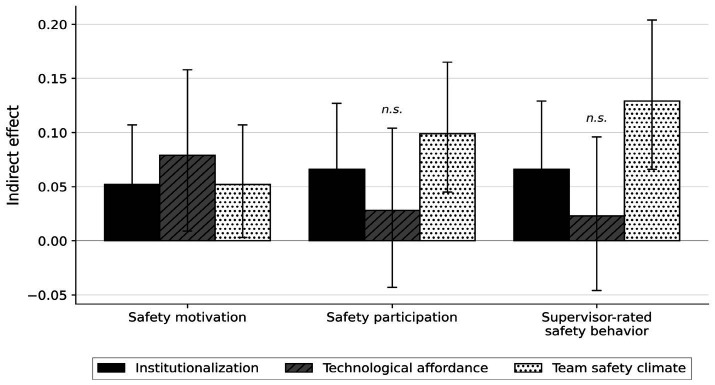
Indirect effect structure showing asymmetric mediation paths. Bars represent specific indirect effects through each organizational mechanism.

The indirect association through technological affordance was significant for safety motivation but not for safety participation, mirroring the asymmetric main-effect pattern. H4a and H4c were supported, whereas H4b received partial support. The indirect effect structure is visualized in Figure 2.

### 4.5 Cross-source comparison

Using supervisor-rated behavioral data (366 teams, 183 firms), II (*β* = 0.303, *p* < 0.01) and SC between-firm (*β* = 0.292, *p* < 0.001) were significantly associated with supervisor-rated behavior, while TE was not (*β* = 0.051, *p* > 0.10). This cross-source pattern is consistent with the self-rated outcome models, although same-source measurement concerns cannot be fully ruled out.

### 4.6 Curvilinear moderation

Finally, this study tested the curvilinear boundary role of performance pressure for the institutional and technological pathways. Table 8 summarizes the moderation estimates.

**Table 8:** Curvilinear moderation by performance pressure.

Variable	SP (Institutional)	SP (Technological)
Mechanism (centered)	0.455*** (0.073)	0.273*** (0.064)
PP (centered)	0.575*** (0.048)	0.615*** (0.048)
PP^2^	−0.138*** (0.040)	−0.163*** (0.042)
Mechanism × PP	0.276*** (0.044)	0.291*** (0.049)
Mechanism × PP^2^	−0.176*** (0.034)	−0.124*** (0.045)
Estimated turning point	≈ 3.47	≈ 3.86

**p < 0.01, ***p < 0.001. Turning points are model estimates on the original 1–5 scale and should be interpreted as approximate thresholds rather than precise managerial benchmarks. Complete coefficient sets for all control variables in the moderation models are reported in Supplementary Table S4.

**Figure 3:**
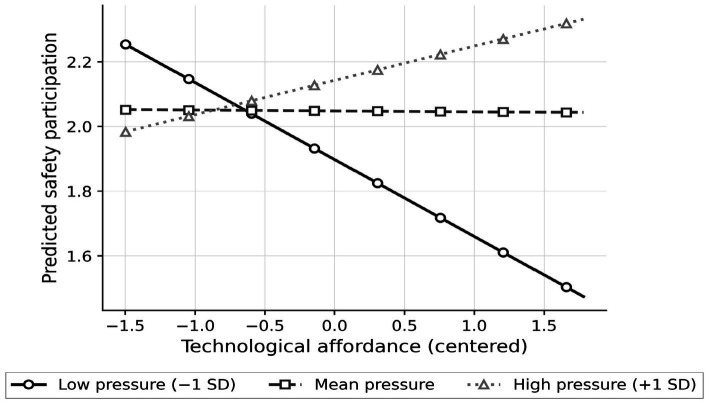
Conditional effect of technological affordance on safety participation at low (−1 SD), mean, and high (+1 SD) levels of performance pressure.

**Figure 4:**
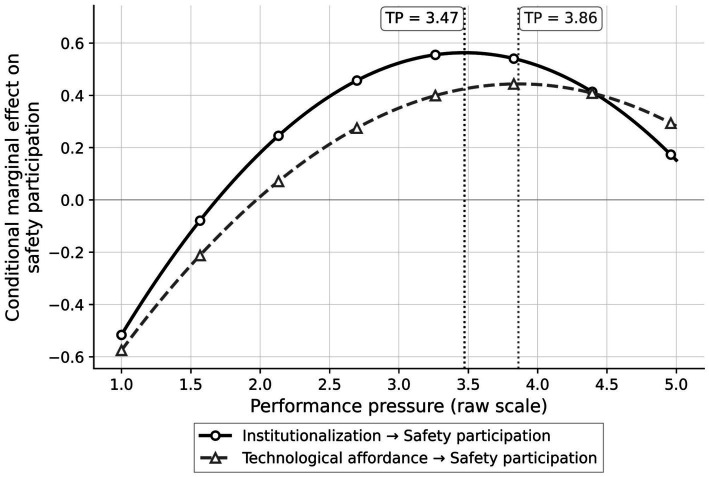
Curvilinear pressure boundaries of behavioral translation. Vertical dashed lines mark estimated inverted-U turning.

H5a and H5b were supported. Figure 3 presents the simple slope analysis for the technological affordance pathway, showing how the conditional effect of technological affordance on safety participation shifts from positive to negligible as performance pressure increases from low to high levels. Figure 4 makes the inverted-U form explicit for both pathways, with the estimated turning points marked on the original scale.

### 4.7 Robustness checks

To determine whether the central asymmetry was robust rather than threshold-dependent, this study subjected the core models to a stricter stress test. This study progressively tightened the minimum team-size requirement and re-estimated the models across alternative aggregation thresholds. Across specifications, the same qualitative pattern held: institutionalization continued to predict both outcomes, whereas technological affordance consistently predicted safety motivation but not safety participation.

The threshold analyses retained 667, 597, 551, 488, and 420 teams across the ≥4, ≥8, ≥10, ≥12, and ≥15 minimum team-size thresholds, respectively; the full coefficient pattern is reported in Supplementary Table S5 and visualized in Supplementary Figure S1.

This study also estimated a simplified random-intercept MSEM as a model-specification check. The MSEM showed acceptable fit (*χ*^2^/df = 2.34, CFI = 0.931, RMSEA = 0.052) and reproduced the core pattern. Technological affordance was associated with safety motivation (*β* = 0.148, SE = 0.052, *p* = 0.004) but not with safety participation (*β* = 0.032, SE = 0.052, *p* = 0.539). Institutionalization was associated with both safety motivation (*β* = 0.244, SE = 0.056, *p* < 0.001) and safety participation (*β* = 0.302, SE = 0.056, *p* < 0.001). Team safety climate was also associated with both safety motivation (*β* = 0.104, SE = 0.041, *p* = 0.011) and safety participation (*β* = 0.224, SE = 0.041, *p* < 0.001). This study therefore treats the MSEM as a robustness supplement, with segmented HLM retained as the primary estimation strategy.

## 5 Discussion

### 5.1 The attitude–behavior gap at the organizational level

The results are consistent with the view that managerial cognitive prioritization does not project directly onto frontline outcomes, but instead operates through heterogeneous organizational mechanisms that relate differently to motivation and behavior. Once the three mechanisms entered the model, the direct association between managerial safety cognition and both frontline outcomes fell to non-significance—a pattern more consistent with organizational mediation than with the direct-signaling logic that underlies much of the safety leadership literature (Zohar, 2002; Clarke, 2013).

This pattern may extend the attention-based view (Ocasio, 1997) in a direction that has received limited empirical attention. Prior applications of the framework have emphasized the *selectivity* of managerial attention—what managers attend to, and what they neglect. Our results suggest that attention distribution may also be *asymmetric* in its downstream consequences: the same cognitive priority, embedded in multiple organizational structures, may cross the threshold from willingness to action through some structures but not through others. The distinction matters because motivational readiness and costly behavioral enactment impose different psychological demands on frontline employees.

These findings sit alongside, but also extend, prior strands of safety research. Consistent with the safety leadership literature (Clarke, 2013; Jiang et al., 2024), our results affirm that managerial-level antecedents matter for frontline safety. This convergence is further reinforced by recent meta-analytic syntheses (Lyubykh et al., 2022; Zhao et al., 2022) and by multilevel evidence tracing how team-level safety leadership shapes frontline behavior through individual safety knowledge (Piao and Hahn, 2025). Consistent with safety motivation work (Griffin and Neal, 2000; Neal and Griffin, 2006), motivation and participation behave as conceptually distinct outcomes. The present findings extend this body of work by relocating the proximal driver from observable leadership behavior to managerial cognitive prioritization, and by separating organizational mechanisms whose translation strength differs across the two outcomes. They differ from accounts that treat safety climate (Zohar, 2000; Zohar and Luria, 2005) or safety commitment as a single dominant pathway: in our data, climate, institutionalization, and technological affordance jointly mediate the link, with technology selectively reaching motivation but stalling at participation.

### 5.2 Why technology may awaken willingness but not action

The most notable finding is the asymmetric pattern of technological affordance: it was consistently associated with SM but not with SP, a pattern replicated across employee self-ratings, supervisor ratings, and all sample threshold conditions. This study offers a theoretically plausible interpretation through two complementary psychological lenses.

First, cognitive load theory (Sweller, 1988) may help explain why technology succeeds at the motivational level. Digital safety systems can reduce the extraneous cognitive load associated with risk identification by converting hidden hazards into visible cues (Cagno et al., 2024), lowering the informational threshold for safety engagement and potentially enhancing safety self-efficacy (Bandura, 1997). This cognitive facilitation may be sufficient to shift motivational readiness.

Second, the interpersonal cost perspective (Detert and Edmondson, 2011) may explain why technology is less effective at the behavioral level. Proactive safety behaviors such as peer intervention, work stoppage, and hazard escalation carry substantial interpersonal costs—role conflict, reputational risk, social anxiety, and relational tension—that technological interfaces are unlikely to eliminate on their own. Instead, overcoming these barriers may require the support of institutional legitimacy, which defines challenging unsafe practice as expected in-role behavior (Morrison, 1994), and psychological safety, which lowers perceived interpersonal risk (Edmondson, 1999). It is important to note, however, that this account remains a theoretically grounded interpretation of the observed pattern rather than a confirmed mechanism. Interpersonal cost, role conflict, social anxiety, and psychological safety risk are theoretically inferred constructs in our argument, and none of them was directly measured in the present study. The interpretation aligns with established work on voice and silence in organizations (Edmondson, 1999; Detert and Edmondson, 2011), and future studies should test this proposition directly by incorporating measures of perceived social cost of voice alongside cognitive load indicators.

Several alternative explanations also deserve attention. Our measurement of technological affordance may capture perceived information accessibility more than actual usage intensity. As Leonardi (2011) noted, the affordances that a technology offers in principle and the ways in which users appropriate those affordances in practice are analytically distinct—a distinction that our cross-sectional perceptual measures do not resolve. In addition, safety participation may be constrained by formal authorization levels not captured in our model, and some high-risk behaviors may require structured voice channels rather than technological support alone.

The climate decomposition points in the same direction. The between-firm component was associated with both outcomes, whereas team-specific deviations were much less consequential, suggesting that costly participation depends more on organization-wide safety norms than on isolated local positivity.

### 5.3 Pressure boundaries and the JD-R framework

The curvilinear moderation is consistent with the JD-R model’s distinction between challenge and hindrance demands (Bakker and Demerouti, 2007; Bakker et al., 2023) and aligns with systematic evidence that production pressure exerts heterogeneous effects on safety outcomes (Hashemian and Triantis, 2023). Within the low-to-moderate range, performance pressure may have functioned as a challenge demand that increased accountability salience. Beyond the estimated turning points (3.47 for II, 3.86 for TE), pressure may have functioned as a hindrance demand associated with normalization of deviance (Vaughan, 1996) and institutional decoupling (Meyer and Rowan, 1977). These turning points are model-derived estimates on a 1–5 Likert scale and should be interpreted as approximate thresholds rather than precise managerial benchmarks.

To contextualize these estimates, the sample average for performance pressure fell below both estimated turning points, indicating that most participating firms operated in the low-to-moderate range where organizational mechanisms were associated with enhanced behavioral outcomes. The institutional pathway’s estimated turning point of 3.47 corresponds to a pressure level approximately one standard deviation above the sample mean—roughly equivalent to firms facing moderately elevated but not extreme production/profit targets. The technological pathway’s higher turning point of 3.86 suggests that digital tools may tolerate greater pressure before degradation, possibly because technology operates continuously regardless of organizational fatigue, whereas institutional enforcement depends on human monitoring that may deplete under extreme pressure.

From a practical standpoint, the inverted-U pattern suggests that managers must attend to what might be termed “dynamic pressure management.” Existing safety systems—whether institutional or technological—are not pressure-proof. When organizations approach peak production seasons or face tight delivery deadlines, managers should not assume that formal safety systems will autonomously maintain frontline safety behaviors. Instead, during these high-pressure periods, leaders may need to deliberately inject additional relational support—strengthening team safety climate through visible commitment and interpersonal backing—to prevent the normalization of deviance and keep costly safety participation viable.

### 5.4 Theoretical contributions

The article makes three theoretical contributions. First, it clarifies a level-of-analysis problem by linking top-level prioritization to two distinct frontline manifestations without collapsing organizational origins into individual outcomes. Second, it shifts explanation away from a generic “leadership matters” claim and toward the organizational carriers through which managerial priorities travel or stall.

That explanatory shift is most visible in the technology result. Organizations can lower informational and motivational thresholds without necessarily lowering the interpersonal and role-related thresholds required for proactive behavior. The attitude–behavior gap is therefore also organizationally structured by the supports employees receive when action becomes costly.

From an organizational-psychology standpoint, the present findings also speak to the broader literature on proactivity and voice. High-cost safety participation is a special case of proactive behavior under conditions of role ambiguity and interpersonal dependence. Employees must decide not only whether a concern is real, but whether raising it is normatively appropriate, strategically wise, and socially survivable. The same organizational arrangements that support safety participation may therefore have parallels in other domains of constructive voice, such as quality improvement, ethical dissent, and reporting of process failures. In this respect, the manuscript’s contribution is not only topical; it also offers a transferable template for analyzing why organizations often find it easier to secure endorsement than enactment.

The third contribution concerns resilience under pressure. Research on production or performance pressure often treats pressure as uniformly harmful to safety. By documenting an inverted-U-shaped boundary effect, the article suggests a more nuanced view. Moderate pressure may activate the relevance of existing institutional and technological supports, whereas excessive pressure erodes their behavioral usefulness. This does not imply that pressure is desirable in itself. Rather, it shows that the behavioral translation of safety priorities depends on the wider demand environment and that genuinely internalized safety systems display a degree of robustness under manageable strain.

A further theoretical implication concerns the status of organizational mechanisms themselves. Institutionalization, technological affordance, and team safety climate do not simply transmit the same influence at different strengths. They alter different classes of constraints. Institutionalization changes what is legitimate and expected; technological affordance changes what is visible and cognitively manageable; climate changes what is socially tolerable and normatively backed at the local level. This differentiation matters because it links multilevel organizational research with fine-grained psychological accounts of self-regulation and action. People do not move from valuing safety to enacting safety under one generic motivational force. They do so under a bundle of organizationally structured conditions that make certain cognitions salient, certain behaviors legitimate, and certain social risks bearable.

The article also contributes to ongoing debates about construct proliferation in organizational research. It does not claim to have definitively established a wholly independent construct that is empirically separable from all variants of safety commitment. Instead, it shows that a cognition-centered, attentional-prioritization framing yields distinctive analytical leverage when the focal problem is cross-level translation under competing demands. In this sense, the contribution is not merely terminological. It lies in specifying an explanatory angle that highlights resource allocation, embedding, and differential downstream thresholds—issues that are less visible when the analysis begins and ends with behavioral signaling.

### 5.5 Practical implications

The practical implications follow directly from the differentiated translation logic. The first implication is that organizations should not rely on symbolic endorsement or episodic safety messaging as substitutes for upstream prioritization. If safety is to become internally sustained rather than externally enforced, senior managers must repeatedly place it in arenas where scarce resources are allocated: budgeting, strategic review, capital investment, staffing decisions, and performance evaluation. In practice, this means integrating safety into executive scorecards, requiring recurrent review of safety implications in strategic meetings, and signaling through resource choices—not only speeches—that safety remains a decision priority when it conflicts with productivity or delivery pressure.

Governance tools should therefore be matched to the downstream change desired. Technology can awaken general willingness, but costly proactive behavior still requires formal accountability and team-level social support.

The second implication concerns pressure management. Many firms intensify production or delivery demands precisely when they need safety systems to function most reliably. The results suggest that managers should not assume that existing systems will retain their behavioral force automatically under such conditions. When operational pressure rises toward peak periods, organizations may need to increase—not decrease—visible relational support, reinforce procedural legitimacy for interruption and reporting, and monitor whether the use of safety technologies remains facilitative rather than coercive. A dynamic pressure-management approach is therefore essential: one that seeks an activation range in which accountability is sharpened without crossing into a zone where overload undermines the enactment of proactive safety behavior.

For managers, this means that safety investments should be sequenced as well as selected. A firm that invests heavily in digital systems before clarifying procedural authority and social expectations may create a situation in which hazards become highly visible but intervention remains under-enacted. Workers may know more, see more, and even feel more concerned, yet still hold back from action. By contrast, when technology is layered onto an environment in which safety-related intervention is already protected by formal rules and supported by team norms, the same technology is more likely to translate into action rather than frustration. The present findings therefore encourage managers to view digitalization as a multiplier of organizational readiness, not as a substitute for it.

A final managerial implication is evaluative: organizations may need different indicators for motivational penetration and behavioral consolidation. Employee surveys that only ask whether safety is valued can overstate the maturity of the system if workers remain reluctant to intervene when intervention is costly. Managers should therefore consider pairing climate or motivation measures with indicators of enacted participation, such as voluntary hazard reporting, peer correction, work stoppage decisions, and upward escalation of safety concerns. Interpreted together, such indicators can reveal whether the organization has merely cultivated endorsement or has genuinely built the institutional and relational conditions under which endorsement becomes action.

A related diagnostic implication concerns digital transformation. Firms often treat the installation of digital systems as evidence that safety governance has become more mature, but digital traceability and standardized reporting can improve the informational environment without necessarily changing the social and institutional consequences of taking action. Organizations undergoing digitalization should therefore assess not only whether systems are in place and used, but whether escalation, interruption, and peer-correction behaviors actually increase.

### 5.6 Limitations and future directions

First, despite the three-wave, multi-source design, this study remains observational. The three-month interval is well-suited to detect proximate translation patterns linking managerial cognition to frontline outcomes but does not capture long-cycle institutional sedimentation. Longitudinal or quasi-experimental designs would help disentangle the directional dynamics between organizational mechanisms and frontline outcomes.

Second, the present findings rest on a sample drawn entirely from Chinese firms operating under a regulatory environment characterized by state-led safety oversight, comparatively centralized accountability structures, and relatively collectivist work-team norms. These institutional features may strengthen the institutionalization pathway by reinforcing the legitimacy of formal safety procedures, and they may shape how frontline employees read team safety climate as a signal of shared expectations. The asymmetric pattern—particularly the weaker behavioral translation through technological affordance—may therefore unfold differently in liberal-market or lower-deference contexts. Cross-cultural replication in firms operating under more decentralized regulatory regimes would help establish the boundary conditions of the present framework.

Third, a related external-validity caveat concerns within-sample heterogeneity across the high-risk industry sectors represented in the data. Manufacturing, construction, mining, and chemical operations differ substantially in hazard exposure profiles, capital intensity, workforce mobility, and the visibility of safety-relevant work routines. Although industry sector was included as a firm-level control, the sample did not permit fully separate sector-stratified estimation. The relative importance of institutionalization, technological affordance, and team safety climate may vary across sectors—for example, technological affordance may operate differently in capital-intensive process industries than in labor-intensive task-based industries. Sector-stratified replication with larger industry-balanced samples would clarify whether the asymmetric mechanism pattern generalizes uniformly across high-risk industry types.

## 6 Conclusion

This study provides evidence that top-level safety prioritization reaches the frontline through differentiated organizational mechanisms rather than through direct projection. Institutionalization and team safety climate were associated with both safety motivation and safety participation, whereas technological affordance was associated only with safety motivation.

Organizations should therefore not treat digital safety technology as a substitute for institutional safeguards or relational support. If the goal is stronger frontline action rather than motivation alone, formal accountability structures and team-level social support remain especially important.

The broader implication is that the path from external compliance to internally sustained safety is neither automatic nor homogeneous. Some mechanisms chiefly awaken willingness; others help convert willingness into action. The enduring challenge of safety governance is therefore not only to make hazards visible, but also to make protective action legitimate, supported, and feasible across organizational levels.

This matters beyond the immediate context of occupational safety. Many organizational priorities—ethics, quality, sustainability, speaking up, inclusion—face a similar translation problem. Senior leaders can value them sincerely, communicate them repeatedly, and invest in visible tools, yet still find that frontline action remains uneven. The present study suggests that the missing link often lies in the architecture of embedding. Priorities become durable only when organizations build mechanisms that address informational, procedural, and interpersonal thresholds simultaneously. Safety is the empirical context here, but the underlying cross-level logic is likely to travel more widely across organizational domains in which willingness is easier to secure than action.

## Data Availability

The raw data supporting the conclusions of this article will be made available by the authors, without undue reservation.
