# Supplementary material for: When technology awakens willingness but stalls action: the asymmetric psychological translation of managerial safety cognition
Source: Front Psychol. 2026 Jun 12;17:1841993. doi: 10.3389/fpsyg.2026.1841993 (PMC13303193; doi:10.3389/fpsyg.2026.1841993)
Supplement: Supplementary file 1 [file Supplementary_file_1.DOCX]

**Supplementary Material**

**Manuscript: *When Technology Awakens Willingness but Stalls Action: The Asymmetric Psychological Translation of Managerial Safety Cognition***

This supplementary file contains the full measurement items (Appendix A), the control-variable codebook (Supplementary Note 1), complete coefficient tables including all control variables (Tables S1–S4), robustness results across alternative aggregation thresholds and high-agreement subsamples (Tables S5 and S9), coefficient-stability results (Figure S1), variance inflation factors (Table S6), discriminant-validity diagnostics (Table S7), and the sample-flow summary (Table S8).

**Supplementary Appendix A. Measurement Items**

*This appendix provides the full item wordings referenced in the main manuscript.*

**Managerial Safety Cognition (Fruhen et al., 2014, 2019; senior managers, T1)**

I consider safety to be as important as production and profitability.

I actively seek information about potential safety hazards.

I ensure that safety is a regular topic in strategic meetings.

When safety conflicts with production targets, I prioritize safety.

I allocate sufficient financial resources to safety improvement.

I am personally involved in reviewing serious safety incidents.

I view safety performance as a key indicator of organizational effectiveness.

I regularly communicate safety expectations to all levels.

**Performance Pressure (adapted from Bakker and Demerouti, 2007; senior managers, T1)**

Our company faces significant pressure to meet profit targets.

Cost reduction requirements create substantial constraints.

Delivery schedules impose strong time pressure on production.

Competitive market conditions require continuous efficiency optimization.

**Institutionalization (adapted from Naveh et al., 2005; safety-function leaders, T2)**

Safety regulations are systematically embedded in standard operating procedures.

Clear accountability mechanisms exist for safety violations at each level.

Safety inspection results are formally linked to performance evaluation.

Written procedures specify employee obligations for hazard reporting.

Regular review cycles ensure safety rules are updated based on incidents.

The accountability loop from identification to corrective action is documented.

**Technological Affordance (adapted from Leonardi, 2011; safety-function leaders, T2)**

Our digital safety systems provide real-time monitoring of key risk indicators.

Technology platforms enable early warning of potential hazards.

Digital tools support standardized safety inspection and reporting.

Safety information systems facilitate data-driven decision-making.

Mobile or on-site devices help employees identify and report hazards.

Technology systems provide feedback closing the hazard-reporting loop.

**Team Safety Climate (adapted from Zohar and Luria, 2005; employees, T2)**

My supervisor emphasizes safety even under deadline pressure.

In our team, safety is considered a priority in daily operations.

Team members are encouraged to report hazards without fear of blame.

Safety issues raised by team members are addressed promptly.

My coworkers care about each other’s safety on the job.

Cutting corners on safety is not accepted in our team.

Our team regularly discusses how to improve safety practices.

Safety rules are followed consistently even without supervision.

**Safety Motivation (adapted from Griffin and Neal, 2000; employees, T3)**

*Note.* Item 3 refers to proactive identification, which may partly tap behavioral intention rather than pure motivational orientation. This overlap is inherent in the Griffin and Neal (2000) framework and does not affect the aggregated construct’s discriminant validity, as confirmed by the two-factor MCFA reported in Section 4.1.

I feel it is worthwhile to put in effort to maintain safety standards.

I believe that safety at work is a very important issue.

I feel it is important to proactively identify safety hazards.

I am motivated to engage in safety activities even when not required.

**Safety Participation (adapted from Neal and Griffin, 2006; employees, T3)**

I voluntarily report safety hazards or near-miss incidents.

I remind coworkers when I notice unsafe practices.

I actively participate in safety meetings and training sessions.

I suggest improvements to safety procedures based on experience.

I would stop work if I believed a serious safety risk existed.

I help new colleagues understand and follow safety procedures.

I raise safety concerns with management even when inconvenient.

I take action to correct unsafe conditions in my work area.

**Supplementary Note 1. Control-variable codebook and provenance**

The table below documents the firm-level control variables used in all analyses. Each row reports the variable code, its survey source and wave, its substantive interpretation, and its coding form. The four B_FACT controls are factual verification items recorded by the safety department at T2 and enter the model specifications as separate standardized firm-level contextual covariates rather than as summary PCA-derived components.

| **Code(s)** | **Source / wave** | **Verified interpretation** | **Notes** |
| --- | --- | --- | --- |
| A_CTRL1 | T1 executive survey | Industry sector | Categorical (1–8); standardized in regression tables. |
| A_CTRL2 | T1 executive survey | Ownership type | Categorical (1–5); standardized in regression tables. |
| A_CTRL3 | T1 executive survey | Firm founding year (firm age source) | Numeric year; standardized in regression tables. |
| A_CTRL4 | T1 executive survey | Workforce size category | Ordinal (1–5); standardized in regression tables. |
| A_CTRL5 | T1 executive survey | Annual revenue category | Ordinal (1–5); standardized in regression tables. |
| A_CTRL6 | T1 executive survey | Risk-level classification | Ordinal (1–4); standardized in regression tables. |
| A_CTRL7 | T1 executive survey | Group-affiliation status | Binary (0/1); standardized in regression tables. |
| A_CTRL8 | T1 executive survey | Recent accident history (past 3 years) | Binary (0/1); standardized in regression tables. |
| A_CTRL9 | T1 executive survey | Safety-investment ratio | Ordinal (1–5); standardized in regression tables. |
| A_CTRL10 | T1 executive survey | Baseline digitalization level | Ordinal (1–5); standardized in regression tables. |
| B_FACT1 | T2 safety-department factual verification | Whether a near-miss incident log was established | Binary indicator (0/1); standardized in regression tables. |
| B_FACT2 | T2 safety-department factual verification | Whether a management-of-change system was established | Binary indicator (0/1); standardized in regression tables. |
| B_FACT3 | T2 safety-department factual verification | Whether high-risk operation approval left a documented trail | Binary indicator (0/1); standardized in regression tables. |
| B_FACT4 | T2 safety-department factual verification | Number of safety-system revisions in the past 12 months | Count; standardized in regression tables. |

*Note. The coefficient tables in Supplementary Tables S1–S4 retain the corresponding A_CTRL1_z–A_CTRL10_z and B_FACT1_z–B_FACT4_z entries consistently across all model specifications.*

**Supplementary Table S1. Full firm-level antecedent models (standardized coefficients)**

| **Variable** | **II antecedent** | **TE antecedent** | **SC antecedent** |
| --- | --- | --- | --- |
| MC_z | 0.220*** (0.060) | 0.437*** (0.073) | 0.451*** (0.089) |
| A_CTRL1_z | −0.038 (0.083) | −0.013 (0.084) | −0.260* (0.105) |
| A_CTRL2_z | 0.035 (0.046) | 0.104* (0.053) | 0.028 (0.070) |
| A_CTRL3_z | −0.047 (0.057) | −0.010 (0.065) | 0.012 (0.078) |
| A_CTRL4_z | 0.139 (0.097) | −0.093 (0.109) | 0.051 (0.149) |
| A_CTRL5_z | −0.037 (0.099) | −0.063 (0.108) | −0.005 (0.149) |
| A_CTRL6_z | −0.075 (0.077) | −0.092 (0.079) | −0.222* (0.101) |
| A_CTRL7_z | −0.061 (0.047) | −0.026 (0.061) | 0.031 (0.075) |
| A_CTRL8_z | 0.021 (0.053) | −0.019 (0.064) | −0.001 (0.074) |
| A_CTRL9_z | −0.011 (0.052) | 0.075 (0.060) | −0.022 (0.074) |
| A_CTRL10_z | 0.053 (0.046) | 0.183** (0.056) | −0.037 (0.063) |
| B_FACT1_z | 0.262*** (0.055) | 0.143* (0.068) | 0.035 (0.077) |
| B_FACT2_z | 0.351*** (0.058) | −0.009 (0.071) | −0.013 (0.088) |
| B_FACT3_z | −0.032 (0.053) | 0.409*** (0.059) | 0.096 (0.070) |
| B_FACT4_z | 0.167** (0.052) | −0.035 (0.057) | −0.021 (0.079) |

*Note.* Cells report standardized coefficients with robust standard errors in parentheses. Models estimated with HC3 heteroscedasticity-robust standard errors at the firm level (*N* = 183). * *p* < 0.05, ** *p* < 0.01, *** *p* < 0.001.

**Supplementary Table S2. Full random-intercept models for safety motivation and safety participation**

| **Variable** | **SM total** | **SM mechanism** | **SP total** | **SP mechanism** |
| --- | --- | --- | --- | --- |
| MC_z | 0.252*** (0.070) | 0.061 (0.081) | 0.239** (0.074) | 0.028 (0.084) |
| II_z | — | 0.234** (0.088) | — | 0.296** (0.091) |
| TE_z | — | 0.179* (0.081) | — | 0.064 (0.084) |
| SC_between_z | — | 0.136* (0.065) | — | 0.261*** (0.066) |
| SC_within_z | — | −0.018 (0.023) | — | 0.042* (0.020) |
| A_CTRL1_z | −0.115 (0.081) | −0.073 (0.078) | −0.125 (0.086) | −0.058 (0.081) |
| A_CTRL2_z | 0.064 (0.052) | 0.035 (0.051) | 0.040 (0.054) | 0.009 (0.052) |
| A_CTRL3_z | 0.022 (0.060) | 0.030 (0.056) | −0.048 (0.063) | −0.023 (0.058) |
| A_CTRL4_z | −0.091 (0.100) | −0.143 (0.096) | −0.053 (0.106) | −0.116 (0.099) |
| A_CTRL5_z | 0.050 (0.099) | 0.064 (0.095) | −0.013 (0.105) | 0.006 (0.098) |
| A_CTRL6_z | −0.098 (0.082) | −0.044 (0.080) | −0.105 (0.087) | −0.020 (0.082) |
| A_CTRL7_z | 0.004 (0.056) | 0.015 (0.053) | −0.009 (0.059) | 0.010 (0.055) |
| A_CTRL8_z | 0.044 (0.060) | 0.029 (0.057) | 0.030 (0.064) | 0.017 (0.059) |
| A_CTRL9_z | 0.022 (0.059) | 0.015 (0.056) | −0.025 (0.062) | −0.020 (0.058) |
| A_CTRL10_z | 0.045 (0.059) | 0.005 (0.058) | −0.019 (0.062) | −0.037 (0.060) |
| B_FACT1_z | 0.195** (0.063) | 0.110 (0.065) | 0.143* (0.067) | 0.054 (0.067) |
| B_FACT2_z | 0.081 (0.072) | −0.001 (0.076) | 0.164* (0.076) | 0.062 (0.078) |
| B_FACT3_z | 0.102 (0.065) | 0.023 (0.071) | 0.003 (0.068) | −0.038 (0.072) |
| B_FACT4_z | −0.101 (0.064) | −0.132* (0.063) | −0.102 (0.068) | −0.146* (0.065) |

*Note.* Cells report standardized coefficients with standard errors in parentheses. Random-intercept mixed models with teams nested within firms. * *p* < 0.05, ** *p* < 0.01, *** *p* < 0.001.

**Supplementary Table S3. Full cross-source models for supervisor-rated safety behavior**

| **Variable** | **SRB total** | **SRB mechanism** |
| --- | --- | --- |
| MC_z | 0.193** (0.074) | −0.029 (0.088) |
| II_z | — | 0.303** (0.097) |
| TE_z | — | 0.051 (0.088) |
| SC_between_z | — | 0.292*** (0.069) |
| SC_within_z | — | 0.028 (0.038) |
| A_CTRL1_z | −0.250** (0.094) | −0.161 (0.089) |
| A_CTRL2_z | 0.049 (0.061) | 0.025 (0.057) |
| A_CTRL3_z | 0.026 (0.065) | 0.037 (0.060) |
| A_CTRL4_z | −0.155 (0.112) | −0.201 (0.104) |
| A_CTRL5_z | 0.041 (0.113) | 0.054 (0.104) |
| A_CTRL6_z | −0.200* (0.092) | −0.108 (0.086) |
| A_CTRL7_z | −0.014 (0.061) | −0.004 (0.056) |
| A_CTRL8_z | 0.062 (0.065) | 0.057 (0.059) |
| A_CTRL9_z | −0.071 (0.061) | −0.066 (0.057) |
| A_CTRL10_z | −0.045 (0.062) | −0.059 (0.059) |
| B_FACT1_z | 0.132* (0.066) | 0.043 (0.065) |
| B_FACT2_z | 0.169* (0.076) | 0.069 (0.077) |
| B_FACT3_z | 0.003 (0.068) | −0.035 (0.071) |
| B_FACT4_z | −0.089 (0.067) | −0.131* (0.064) |

*Note.* Cells report standardized coefficients with standard errors in parentheses. Random-intercept mixed models estimated on the supervisor-rated subsample (366 teams in 183 firms). * *p* < 0.05, ** *p* < 0.01, *** *p* < 0.001.

**Supplementary Table S4. Full curvilinear moderation models (standardized coefficients)**

| **Variable** | **Institutional pathway** | **Technological pathway** |
| --- | --- | --- |
| II_z | 0.455*** (0.073) | — |
| TE_z | — | 0.273*** (0.064) |
| PPm | 0.575*** (0.048) | 0.615*** (0.048) |
| PPm² | −0.138*** (0.040) | −0.163*** (0.042) |
| II_z × PPm | 0.276*** (0.044) | — |
| II_z × PPm² | −0.176*** (0.034) | — |
| TE_z × PPm | — | 0.291*** (0.049) |
| TE_z × PPm² | — | −0.124*** (0.045) |
| A_CTRL1_z | −0.161* (0.066) | −0.148* (0.066) |
| A_CTRL2_z | 0.013 (0.042) | 0.005 (0.043) |
| A_CTRL3_z | 0.097* (0.045) | 0.038 (0.046) |
| A_CTRL4_z | −0.107 (0.080) | 0.080 (0.080) |
| A_CTRL5_z | 0.078 (0.079) | −0.005 (0.079) |
| A_CTRL6_z | −0.070 (0.065) | −0.067 (0.066) |
| A_CTRL7_z | −0.013 (0.043) | −0.046 (0.042) |
| A_CTRL8_z | 0.036 (0.045) | 0.033 (0.045) |
| A_CTRL9_z | −0.037 (0.042) | −0.050 (0.042) |
| A_CTRL10_z | −0.014 (0.043) | −0.030 (0.044) |
| B_FACT1_z | −0.005 (0.049) | 0.004 (0.048) |
| B_FACT2_z | 0.067 (0.057) | 0.166** (0.052) |
| B_FACT3_z | 0.056 (0.046) | −0.034 (0.055) |
| B_FACT4_z | −0.097* (0.048) | 0.010 (0.047) |

*Note.* PPm denotes grand-mean-centered performance pressure; PPm² denotes its squared term. Cells report standardized coefficients with standard errors in parentheses. * *p* < 0.05, ** *p* < 0.01, *** *p* < 0.001.

**Supplementary Table S5. Robustness: coefficient stability across team-size thresholds**

| **Threshold** | **DV** | **N teams** | **N firms** | **II** | **TE** | **SC (between)** | **SC (within)** |
| --- | --- | --- | --- | --- | --- | --- | --- |
| ≥ 4 | SM | 667 | 183 | 0.234 | 0.179 | 0.136 | −0.018 |
| ≥ 4 | SP | 667 | 183 | 0.296 | 0.064 | 0.261 | 0.042 |
| ≥ 8 | SM | 597 | 173 | 0.250 | 0.237 | 0.170 | −0.024 |
| ≥ 8 | SP | 597 | 173 | 0.299 | 0.075 | 0.335 | 0.040 |
| ≥ 10 | SM | 551 | 166 | 0.213 | 0.243 | 0.160 | −0.010 |
| ≥ 10 | SP | 551 | 166 | 0.296 | 0.102 | 0.323 | 0.053 |
| ≥ 12 | SM | 488 | 148 | 0.227 | 0.295 | 0.140 | 0.001 |
| ≥ 12 | SP | 488 | 148 | 0.298 | 0.129 | 0.332 | 0.078 |
| ≥ 15 | SM | 420 | 130 | 0.215 | 0.287 | 0.154 | 0.006 |
| ≥ 15 | SP | 420 | 130 | 0.299 | 0.116 | 0.380 | 0.087 |
| Supervisor | SRB | 366 | 183 | 0.303 | 0.051 | 0.292 | 0.036 |

*Note.* Coefficient pattern remains substantively stable across alternative aggregation thresholds and the supervisor-rated specification.

**Supplementary Figure S1. Coefficient stability across team-size thresholds**


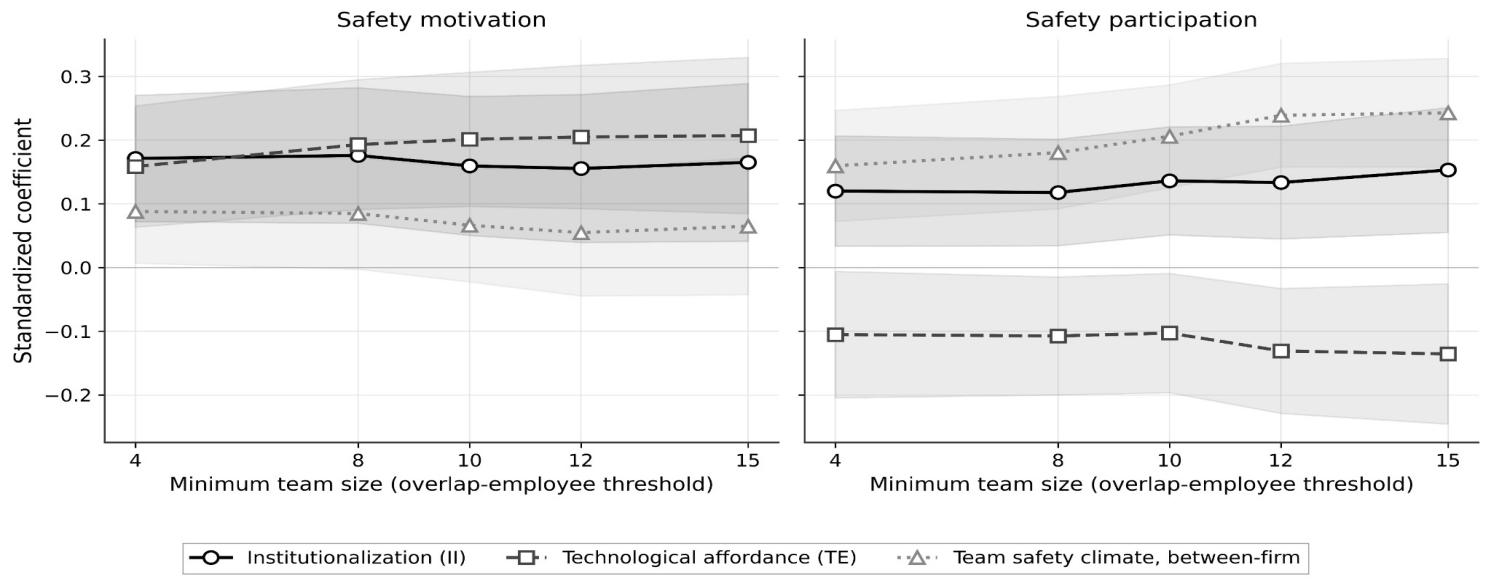


Lines show the standardized coefficients for institutionalization, technological affordance, and the between-firm component of team safety climate across alternative minimum team-size thresholds.

**Supplementary Table S6. Variance inflation factors for firm-level mechanism predictors**

| **Predictor** | **VIF** |
| --- | --- |
| Managerial safety cognition (MC) | 2.05 |
| Institutionalization (II) | 1.44 |
| Technological affordance (TE) | 1.49 |
| Team safety climate (SC, between-firm component) | 1.37 |

*Note.* VIF values are computed for the firm-level mechanism regressions in which managerial safety cognition, institutionalization, technological affordance, and the between-firm component of team safety climate are entered as simultaneous predictors. All values fall well below the conventional 5.0 threshold, indicating that multicollinearity does not pose a problem for the estimation of mechanism path coefficients.

**Supplementary Table S7. Discriminant-validity diagnostics for safety motivation and safety participation**

| **Diagnostic** | **Value** | **Calculation basis** |
| --- | --- | --- |
| HTMT(SM, SP), individual item-level | 0.665 | Raw individual employee item responses, *N* = 12,379 |
| HTMT(SM, SP), full team-aggregated | 0.779 | Item means at team level, *N* = 667 |
| HTMT(SM, SP), firm-aggregated | 0.834 | Item means at firm level, *N* = 183 |
| HTMT(SM, SP), supervisor-rated subsample team-level | 0.767 | Item means at team level, *N* = 366 |
| Pearson r(SM, SP), individual level | 0.635 | Scale scores, *N* = 12,379 |
| Disattenuated r(SM, SP) | 0.666 | Based on scale-score correlation and reliability estimates |
| Fornell–Larcker √AVE_SP | 0.904 | Item-level CFA |
| Fornell–Larcker √AVE_SM | 0.919 | Item-level CFA |
| Latent inter-factor correlation SM–SP | 0.730 | Item-level CFA |

*Note.* All HTMT estimates fall below the conservative 0.85 threshold (Henseler et al., 2015) regardless of aggregation level, supporting the interpretation that the higher firm-aggregated descriptive correlation between SM and SP (*r* = 0.829, see main manuscript Table 5) is consistent with aggregation-based attenuation of random measurement error rather than overlap of underlying constructs. The Fornell–Larcker √AVE values are computed at the item level on individual employee responses (*N* = 12,379) and should not be directly compared with the AVE values reported in the main manuscript’s Table 4, which are based on the manuscript’s CFA measurement model.

**Supplementary Table S8. Sample flow across the three-wave data collection**

| **Stage** | **Unit / source** | **Firms** | **Teams** | **Individual responses** |
| --- | --- | --- | --- | --- |
| T1 field sample | Senior managers | 312 | — | — |
| T2 field-stage matched sample (pre-final) | Safety department heads + frontline employees | 237 | 892 | — |
| Final matched analytic sample (firms) | Firms retained after invalid, missing, and unmatched records were removed | 183 | — | — |
| T2 retained sample (final analytic): safety-department survey | Safety department heads | 183 | — | 183 |
| T2 retained sample (final analytic): employee survey | Frontline employees (team safety climate) | 183 | 667 | 11,691 |
| T3 retained sample (final analytic): employee survey | Frontline employees (safety motivation and participation) | 183 | 667 | 12,379 |
| T3 supervisor-rated validation subsample | Direct supervisors | 183 | 366 | 366 |

*Note.* The table reports the auditable field-flow beginning from firms that entered the T1 field sample. The supervisor-rated validation subsample was drawn from all 183 final retained firms but covered 366 of the 667 teams because not all direct supervisors consented to provide independent behavioral ratings. Em dashes indicate that auditable counts are not available at the corresponding stage. Post-hoc exclusion thresholds—such as response-time cutoffs, straight-lining rules, or attention-check criteria—were not part of the retained field-processing record and are therefore not retrospectively reconstructed.

**Supplementary Table S9. Mechanism regressions in the strict high-agreement subsample**

| **Predictor** | **Safety motivation** | **Safety participation** |
| --- | --- | --- |
| Managerial safety cognition (MC) | 0.000 (0.123) | −0.011 (0.125) |
| Institutionalization (II) | 0.353** (0.123) | 0.356** (0.125) |
| Technological affordance (TE) | 0.245* (0.107) | 0.115 (0.109) |
| Team safety climate (between-firm) | 0.135 (0.101) | 0.299** (0.103) |
| Team safety climate (within-firm) | −0.090* (0.046) | 0.007 (0.042) |

*Note.* The strict subsample retained only teams with *r_wg_* ≥ 0.70 for team safety climate, safety motivation, and safety participation (*N* = 191 teams nested within 122 firms). Cells report standardized coefficients with standard errors in parentheses. Random-intercept mixed models with teams nested within firms; control variables (A_CTRL1–A_CTRL10, B_FACT1–B_FACT4) were included as in the main models. The analysis was conducted to assess whether the mechanism pattern was sensitive to low within-team agreement in frontline employee-rated constructs. * *p* < 0.05, ** *p* < 0.01, *** *p* < 0.001.
